# Exploring the Optimal Clinical Cutoff for the IGT‐AD Distress Subscale Adapted for AD Biomarker Results Disclosure

**DOI:** 10.1002/alz70858_102082

**Published:** 2025-12-25

**Authors:** Dianxu Ren, Jeong Eun Kim, Joshua D Grill, Jennifer H Lingler

**Affiliations:** ^1^ University of Pittsburgh, Pittsburgh, PA, USA; ^2^ School of Nursing, University of Pittsburgh, Pittsburgh, PA, USA; ^3^ University of California, Irvine, Irvine, CA, USA; ^4^ Institute for Memory Impairments and Neurological Disorders, University of California, Irvine, Irvine, CA, USA; ^5^ University of Pittsburgh Alzheimer's Disease Research Center (ADRC), Pittsburgh, PA, USA

## Abstract

**Background:**

The **Impact of Genetic Susceptibility Testing for Alzheimer's Disease (IGT‐AD)** instrument, developed to assess the psychological effects of learning genetic information specific to Alzheimer's disease (AD), has been widely used in the literature. Identifying a cutoff score for this screening instrument is crucial for rapidly and efficiently identifying individuals in need of psychological intervention, including those learning the results of non‐genetic AD biomarker testing. This study aims to explore the optimal cutoff score and evaluate the predictive powers of an adapted IGT‐AD Distress subscale for identifying distress in cognitively symptomatic older adults learning the results of amyloid PET testing.

**Method:**

We adapted the IGT‐AD instrument by replacing reference to genetic testing with reference to biomarker testing. A total of 96 cognitively symptomatic research participants were assessed for psychological impact using this adapted instrument and the **Impact of Event Scale (IES)** as a gold standard comparison diagnostic tool. The optimal cutoff point for the modified IGT‐AD in detecting distress was determined by evaluating the sensitivity and specificity of various cutoff scores, with an IES cutoff of 26 used as the gold standard. Receiver Operating Characteristic (ROC) analysis was performed, and the area under the curve (AUC) was calculated to evaluate the predictive accuracy of the IGT‐AD. The **Youden index** was used to identify the cutoff point that maximized sensitivity and specificity.

**Result:**

The mean age of participants was 72.4 years, 40.6% were male, 71.6% were White race, 21% were Black race, and 20% were Hispanic ethnicity. Most (55.2%) held bachelor's or post‐bachelor's degrees and 71.9% were married or cohabiting. The optimal cutoff point for the modified IGT‐AD Distress subscale was determined to be 22, with a sensitivity of 77.1% and specificity of 82.0%. The AUC for distress was 0.86 (95% CI: 0.78–0.94), indicating excellent predictive power.

**Conclusion:**

The findings suggest that an adapted IGT‐AD Distress subscale score above the identified cutoff of 22 is effective in detecting psychological distress in cognitively symptomatic persons learning their AD biomarker status. This cutoff may have practical applications in clinical and research settings for screening and early intervention.

